# Involvement of Interleukin-1 Receptor-Associated Kinase 4 and Interferon Regulatory Factor 5 in the Immunopathogenesis of SARS-CoV-2 Infection: Implications for the Treatment of COVID-19

**DOI:** 10.3389/fimmu.2021.638446

**Published:** 2021-04-07

**Authors:** Nicholas Stoy

**Affiliations:** Department of Physiology, Anatomy and Genetics, University of Oxford, Oxford, United Kingdom

**Keywords:** COVID-19, IRAK4, IRF5, M1 macrophages, cytokine storm, Pellino-1, innate immunity, adaptive immunity

## Abstract

Interleukin-1 receptor-associated kinase 4 (IRAK4) and interferon regulatory factor 5 (IRF5) lie sequentially on a signaling pathway activated by ligands of the IL-1 receptor and/or multiple TLRs located either on plasma or endosomal membranes. Activated IRF5, in conjunction with other synergistic transcription factors, notably NF-κB, is crucially required for the production of proinflammatory cytokines in the innate immune response to microbial infection. The IRAK4-IRF5 axis could therefore have a major role in the induction of the signature cytokines and chemokines of the hyperinflammatory state associated with severe morbidity and mortality in COVID-19. Here a case is made for considering IRAK4 or IRF5 inhibitors as potential therapies for the “cytokine storm” of COVID-19.

## Introduction

Effective treatments are required for COVID-19 hyperinflammatory syndrome, occurring characteristically 7–14 days after first symptoms (1) and variously described as “macrophage activation syndrome” (2), “cytokine storm” (3) or “acute respiratory distress syndrome” (4). Its immunological hallmarks are excessive elevation of predominantly proinflammatory cytokines, chemokines (5), and other bioactive molecules, such as HMGB1 (6) and reactive oxygen species (7). Upregulated cytokines include IL-6, TNF-α, IFN-γ, IL-1β, IL-15, IL-23, and IL-10, and chemokines, CXCL8(IL-8), CXCL9(MIG), CXCL10(IP10), CCL2(MCP-1), CCL3(MIP-1α), CCL5(RANTES), CCL7(MCP-3), CCL8(MCP-2), CCL11(eotaxin-1), and CCL20(MIP-3α) (1, 2, 8–11). This review examines the role of IRAK4 and IRF5 in the evolution and modulation of the immune response to SARS-CoV-2 and whether IRAK4 or IRF5 inhibitors could have a role in treating the hyperinflammatory phase (12–14).

## Overview of IRAK4 and IRF5 Signaling

**Figure 1:**
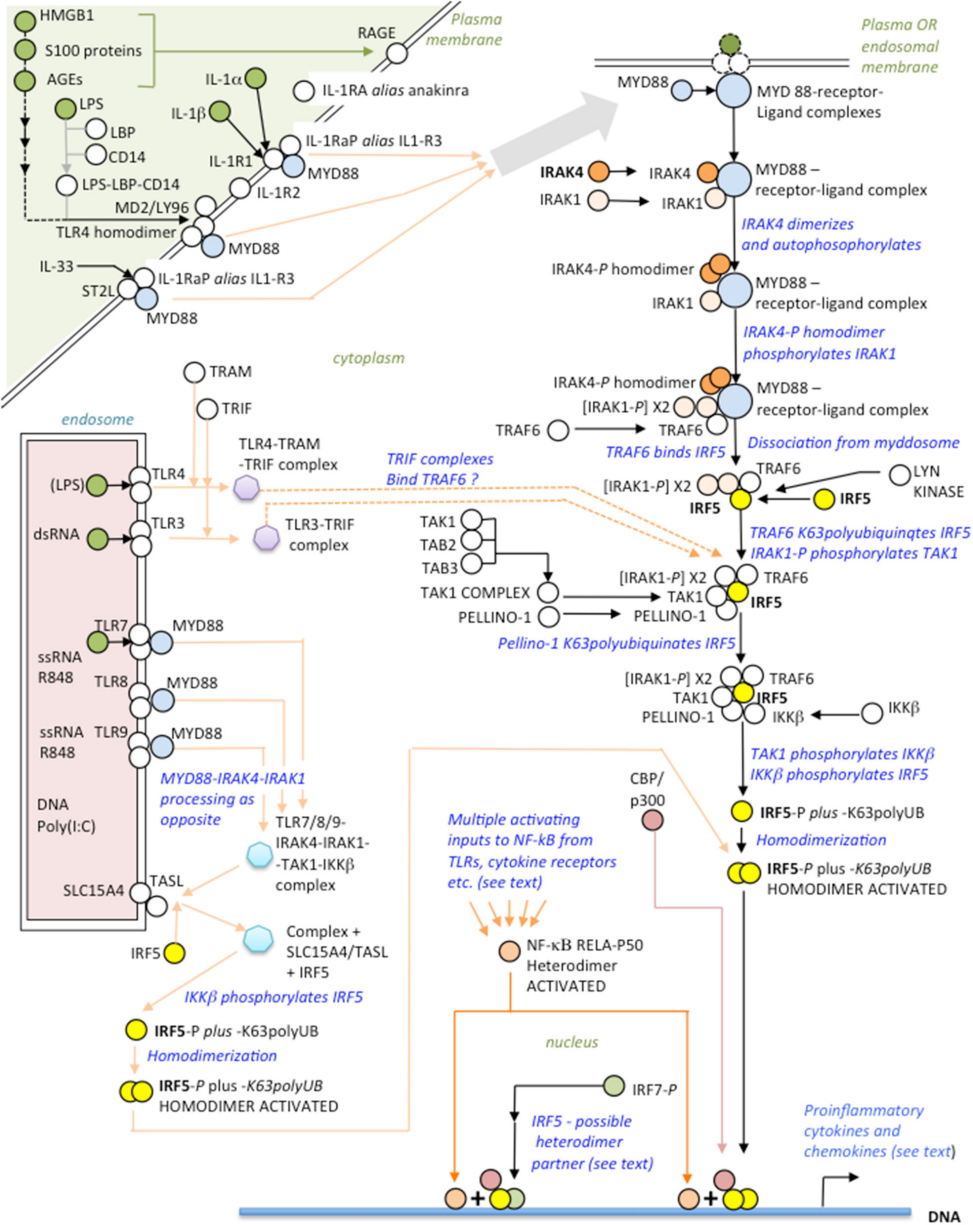
Overview of the IRAK4-IRF5 signaling axis and some ligands of possible relevance to SARS-CoV-2 immunopathogenesis.

IRAK4, recruited with other binding partners to MYD88 (Figure 1) forms the myddosome (14–16), which is activated by ligands of the IL-1 receptor or TLRs that bind MYD88 (13, 17, 18). IRAK4 is recruited to the complex with IRAK1 and TRAF6 (19). On receptor activation IRAK4 homodimerizes, autophosphorylates and subsequently phosphorylates IRAK1 (20–22). These kinases are ultimately responsible for activation of IRF5, requiring phosphorylation of critical C-teminal serines (23, 24). Another component of IRF5 activation is its K63polyubiquitination by TRAF6 (25, 26). The inducible (and IRAK1-phosphorylated) ubiquitin ligase Pellino-1, with E2-conjugating enzymes (27), reciprocally K63polyubiquinates both IRF5 and IRAK1/4 but, conversely, kinase-active IRAK1/4 mediates degradative polyubiquitination of Pellino-1 (27–29).

Distal to the myddosome the signaling pathway bifurcates into IRF5- and NF-κB-activating branches (30). At commencement of the IRF5 branch, activated IRAK4/IRAK1 phosphorylates the kinase TAK1 (31), which in turn phosphorylates IKKβ (24, 32); finally, IKKβ phosphorylates IRF5 (33, 34) facilitating its dimerization and translocation to the nucleus (35). Whereas, IKKβ is the archetypal kinase activator of NF-κB by phosphorylating IκBα, it is important to appreciate that kinase activity of IRAK4 is not essential for NF-κB activation by the myddosome route (35); however, this does not preclude an IRAK4 scaffolding function (20). Crucially, therefore, blocking IRAK4 with a specific kinase inhibitor abolishes IRF5 activation but still permits NF-κB activation by other means, either by IKKβ itself via this or other signaling pathways (31), or using other kinases such as a MEKK3-dependent pathway (17, 30, 35, 36). Speculatively, endosomal TLR3, responsive to dsRNA, may signal independently of MYD88 to IRF5 through TRAF6 using the adaptor TRIF (as well as to IRF3/7 via TRAF3) and may synergise with other TLRs (37, 38, 183); TLR4, translocated to endosomes, may also signal using TRAM-TRIF instead of MYD88 (19, 39–41, 183). IRF5 homodimers complex with CBP/p300 to initiate the IRF5 transcriptome synergistically with NF-κB (42, 43).

Activation of IRF5 is tightly controlled. Inducible IRAK-M inhibits assembly of the IRAK1-IRAK4-TRAF6 complex both directly, and indirectly by induced negative feedback (44–46); Lyn kinase, in dendritic cells (DCs), binds IRF5, inhibiting its K63polyubiquitination and phosphorylation, but not affecting the NF-κB branch (47); IRF8 competes with IRF5 at promoters, blocking its action (48, 49); and KAP1/TRIM28 is an IRF5 transcriptional co-repressor (50). TLRS 7-9 require at least two adapter proteins, TASL and SLC15A4, at the endosomal membrane, to engage the IRAK4-IRF5 pathway (51).

In responding to viruses, activated IRF5 homodimers bind with low affinity to “viral response elements” inducing primarily IFNA type I interferons (52). However, IRF5 binds strongly to the regulatory loci of other IRF5-targetted genes, such as IFNB, CXCL10, IL-10 (52), IL-12, and IL-23 (53), although in the case of anti-inflammatory IL-10, IRF5 is not directly responsible for its elevation in “cytokine storm,” being inhibitory at the IL-10 promoter (53, 54). Mechanistically, a challenging complexity of variables influence IRAK4-IRF5 pathway activation outcomes (55): these include different IRF5 dimerization partners—including homodimerization and IRF7 (56); functionally different IRF5 isoforms, as investigated in plasmacytoid DCs (pDCs) (57); IRF5 interacting with different transcription factors (17), most critically the NF-κB subunits, p50 (48, 58), and/or p65(RELA) (41, 59, 60); different cellular localizations, notably monocytes, macrophages, pDCs, and B cells (55, 58, 61); different triggers of pathway activation, for e.g., viral infection or autoimmunity; inhibition of the IRF5-mediated activation of IFN-β by the IKKα pathway (62); and differences between murine and human cells (63)—all beyond the scope of this review. Nevertheless, despite these many complicating factors, the IRAK4-IRF5 axis consistently polarizes monocytes/macrophages toward the proinflammatory M1 (49, 53, 64) phenotype, displaying a similar innate cytokine/chemokine profile as in “cyokine storm” and indicating a potential therapeutic role for IRAK4 or IRF5 inhibition.

## Deficiency or Inhibition of IRAK4 or IRF5 and Viral Infections

A proinflammatory response is characteristic of the innate immune system's reaction to microbial infection. Endotoxin tolerance in monocytes blunts this response by interfering with recruitment and activation of IRAK4 at the MYD88 receptor complex, inhibiting K63polyubiquitination of IRAK1 and TRAF6, and compromising IRAK1-TRAF6 function and TAK1 activation (65). Mice lacking IRAK4 exhibit deficient IL-1 and TLR signaling, are resistant to LPS and cannot induce TNF-α or IL-6 (17, 66, 67). The IRAK4 inhibitor, chlorogenic acid, extracted from lonicerae flos, protects mice from endotoxic shock: chlorogenic acid inhibits autophosphorylation of IRAK4 in peritoneal macrophages subjected to various activating stimuli, including ssRNA, IL-1α, or HMGB1 (6, 68, 69). Inhibition of IRAK4 or IRF5 downregulates the proinflammatory IRF5 transcriptome independently of NF-κB activation (35). In the same way that endotoxic shock is abrogated by inhibiting IRF5, “cytokine storm” in viral infection can also be suppressed by IRF5 inhibition, as shown for influenza A (26, 70). Thus, IRF5 inhibition protects from hyperinflammation whether induced by viral or bacterial infection, the latter a common complication of acute respiratory distress syndrome, although its incidence in COVID-19 is only just being investigated (71–73, 184).

## The Immunopathogenesis and Clinical Correlates of SARS-CoV-2 Infection

**Figure 2:**
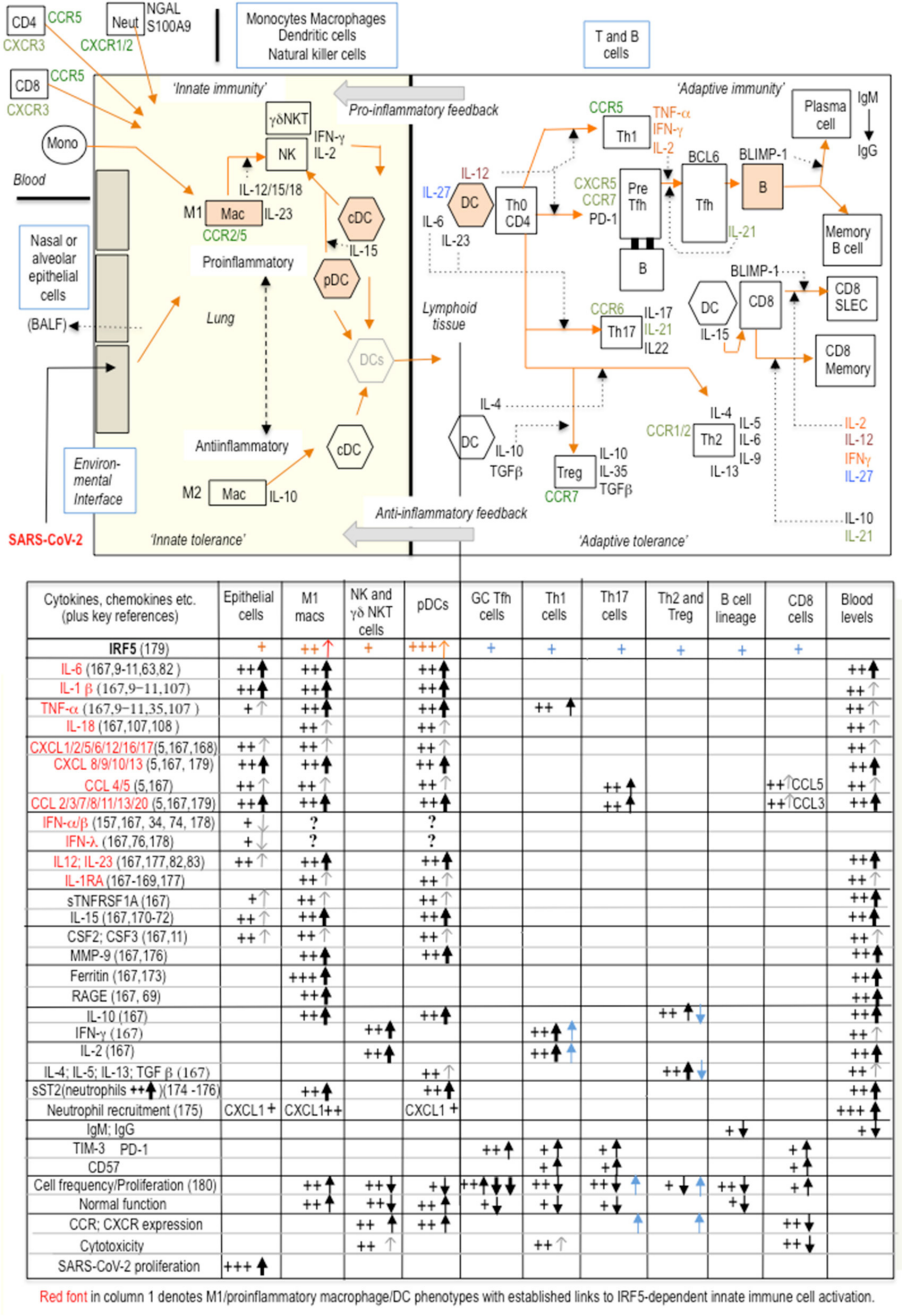
With burgeoning knowledge of immune cell phenotypes in COVID-19, particularly from single-cell transcriptomics, and despite much heterogeneity amongst T-cell clusters, it is now possible to attempt a broad generalization of (at least some) changes in innate and adaptive immunity during COVID-19 progression. In this schematic representation, a suppressed type I interferon response in epithelial cells, IRF5-dependent proinflammatory macrophage and DC polarization, and an inadequate adaptive response are three sequential major drivers of COVID-19 immunopathogenesis. The accompanying table is a tentative interpretation of some of the (sometimes conflicting) features of the immune cell phenotypic landscape of “cytokine storm” (1, 2, 9–11, 157–163). SARS-CoV-2 can be taken up by macrophages and DCs but does not proliferate, whilst the highly variable type I IFN response from each individual cell likely depends on temporal sequencing and integration of inputs both from viral components and from other non-viral inputs (TLR and/or cytokine), either synergistic or separate (179–183). Key: +signs indicate changes in immune cell parameters associated with “cytokine storm” and black vertical arrows indicate changes from the expected normal immune response. IRF5 appears to be widely expressed in most immune cells (164–166) and recently CD4+ and CD8+ intrinsic IRF5 activity has been demonstrated to be responsible for increased secretion of Th1 and Th17 cytokines and for reduced Th2 and T reg cytokines, on T cell activation, as indicated by blue arrows and blue +signs in the table: IRF5 upregulates chemokine receptors CXCR4/5 and CCR6/7/9, on stimulated CD4+ T cells (165, 166); the relevance of these observations to COVID-19 pathogenesis is as yet unknown. SLEC: short-lived effector cells; BALF: bronchoalveolar lavage fluid; GC, germinal center.

Figure 2 summarizes how SARS-CoV-2 innate immune activation is linked to specific T cell (cytotoxic and memory) and B cell (antibody) adaptive immune responses, comprising the substantive immunological reaction necessary for viral elimination (53, 54). COVID-19 immunopathogenesis divides conveniently into three overlapping interactive phases with sequential involvement of epithelial cells, innate immune cells and adaptive immune cells. Nasal and alveolar type II epithelial cells express high levels of ACE2, the SARS-CoV-2 entry receptor, and respond first. Epithelial immune activation is mediated by IRF3 phospho-dimerisation, with a lesser contribution from IRF5 phospho-dimerization, and NF-κB p65 as coactivator (59, 60, 75). Critically, the type I interferon component of the epithelial cell proinflammatory response is selectively suppressed by proliferating SARS-CoV-2 (74), so disrupting secondary expression of interferon-stimulated genes (ISGs) including the potent IRF3 dimerisation partner IRF7 (75). Epithelial cells favor IFN-λ expression but this is a less effective inducer of ISGs than type I interferons (76). The viral MDA5 RNA-sensor requires kinases TBK1 or IKKε to activate IRF3 (77, 78); the same kinases can activate IRF5. By contrast, IKKβ, a strong activator of IRF5, fails to activate IRF3 (61) In phase two, epithelial chemokines attract a large influx of innate immune cells comprising DCs, natural killer (NK) cells and neutrophils (11, 79–81); IRF5 is considered the main orchestrator of this innate response (61). DCs are pivotal in communicating with the adaptive immune system to initiate phase three: programming of adaptive immunity. Phase three culminates either in viral clearance and COVID-19 resolution or complications such as “cytokine storm,” clotting disorders, cardiovascular complications or multi-organ failure. Involvement of adaptive immunity adds further cytokines to the mix. IL-17, the product of Th-17 cells, is triggered by DCs expressing IL-23 (increased with age). Induction of this and other DC cytokines IL-1β, IL-6, IL-12, and TNF-α is again dependent on IRF5, usually with NF-κB coactivators (53, 58, 59, 61, 82–84). Indeed, recent evidence suggests IRF5 may even vie with IRF7 for the title ‘master regulator’, if not of type I interferons, at least of most other DC cytokines (42, 52, 75, 85–88); furthermore, there is mutual inhibition between these two IRFs (56). Th1 cells are activated by the innate cytokine IL-12 from DCs and secrete IFN-γ, as do NK cells. However, in COVID-19 multifunctional activated T cells secreting two of the three cytokines IFN-γ, IL-2, and TNF-α were reduced whilst T cells producing all three were non-functional (89).

Innate immunity is relatively preserved during aging and constitutively upregulated in many comorbid conditions exacerbating COVID-19, albeit stimulating a defective adaptive response. In aging, pDCs retain most of the proinflammatory phenotype, but type I and type III interferons are impaired (90), as are interactions with T and B cells for antigen presentation, primarily due to T and B cell dysfunction, exacerbated by SARS-CoV-2 (91). IRF5 is constitutively expressed by pDCs, especially in females who produce more IFN-α on TLR stimulation than males, making dysregulation of immune responses in COVID-19 in females less likely (92, 93). IRF5-dependent IFN-β expression in DCs is demonstrated in IRF5-knock-out mice, which exhibit poor interferon responses to TLR stimulation or microbial infection (49, 94). Overall, DCs adopt a proinflammatory phenotype on contact with SARS-CoV-2, a tendency exacerbated by increasing age (91, 95). Cellular correlates of poor outcome in COVID-19 are neutrophilia (3), low CD4+ and CD8+ T cells and general lymphopenia (96, 97), combined with increased markers of T cell exhaustion (PD-1 and TIM-3) and senescence (CD57) and a specific cytokine signature (10, 73, 98–100).

B cells depend on IRF5-induced Blimp-1 for differentiation into plasma cells, responsible for long-lasting antibody immunity (101). In SARS-CoV-2 “cytokine storm,” B cell function is compromised by reduced total circulating B cells, reduced class switching from IgM to IgG and increased plasmablasts and transitional cells, suggestive of rapid B cell proliferation and exhaustion, probably related to excessive IL-6 and TNF-α (102).

Platelets are integral components of the immune system. Viruses can enter platelets, activate endosomal TLRs (TLR7/TLR9) and downstream MYD88-IRAK4-IRAK1-IKKβ (and presumably IRF5), possibly contributing to COVID-19 thrombocytopenia and clotting irregularities (103, 104).

## The Metabolic Dimension and COVID-19 Comorbidities

Maintaining the M1 phenotype is energy-consuming and achieved by a “metabolic switch” from oxidative to glycolytic metabolism during M2-to-M1 polarization (41). Viral infections increase glucose metabolism in macrophages, involving activation of the hexosamine biosynthesis pathway and associated enzyme O-GlcNAc transferase, as already proposed for SARS-CoV-2 (105). Thus, increased activation of IRF5 by K63polyubiquitination may turn out to provide an important link between so-called “metabolic inflammation” and increased severity of the cytokine response in COVID-19 (105, 106). Infection with influenza virus markedly increases GlcNAcylation of IRF5 at serine 430 in human macrophages, which is essential for K63polyubiquitination of the same residue that activates IRF5, thus promoting proinflammatory cytokine expression and possibly increased viral replication (26). Inflammation is a well-recognized driver of the metabolic syndrome, manifest clinically in obesity, type II diabetes and other conditions in which insulin resistance occurs. Blood sugar instability associates with IRF5 upregulation and M1 phenotype in adipose tissue macrophages, including elevation of the cardiovascular risk factor, matrix metalloproteinase-9. TLR4 is also upregulated and it has even been hypothesized that increased proinflammatory cytokines could be triggered by endogenous TLR4 ligands, presumably through IRAK4-IRF5 signaling (41). IRF5 knock-out mice exhibit improved glucose tolerance and reduced excess body fat. The M1 macrophage cytokine and chemokine profile of adipose tissue in obesity and diabetes, signifying chronic inflammation, is in many respects similar to a muted version of cytokine storm. Thus, adipose IRF5 transcripts in obesity correlate positively with TNF-α, IL-1β, IL-6, CXCL8/IL-8, CXCL9/MIG, CXCL10/IP10, CCL2/MCP1, CCL5, and CCL7/MCP3, all of which can be elevated in COVID-19 “cytokine storm” (107, 108); positive correlations with IL-2 and IL-12 have been reported by the same group. TLR4, TLR7, and TLR8 are increased in obesity and correlate with IRF5 expression, but whether this occurs in SARS-CoV-2 infection is unknown (109, 110).

There is increased Pellino-1 expression in adipose tissue macrophages in obesity. Pellino-1 exacerbates glucose intolerance in obese mice through K63polyubiquitination of IRF5, promoting M1 macrophage polarization (106); the adverse proinflammatory skewing of innate immunity is further compounded by Pellino-1 inhibition of tolerogenic M2 macrophages by K63polyubiquitination of IRAK1 (111). Correspondingly, in acute viral respiratory infections, there is an association between elevated Pellino-1 and proinflammatory cytokines (112).

Chronic innate proinflammatory drive to the adaptive immune system in metabolic inflammation leads eventual to T cell exhaustion. Changes in T and B cell function in metabolic inflammation, as well as in the elderly and, more acutely, in COVID-19 are all broadly similar, in that innate function is relatively preserved, but T cell and B cell compartments exhibit features of “exhaustion” or “senescence” (73, 113, 114). Therefore, pre-existing metabolic inflammation across a variety of chronic conditions presages an unfavorable course and outcome of COVID-19 (115, 116).

## Discussion

The well-established paradigm that innate immunity programs adaptive immunity applies not only in microbial infection (117) but also autoimmunity (118, 119) and cancer, being generally tolerogenic in the latter. “Cytokine storm” of COVID-19 illustrates the dangers of a fundamental mismatch between increased proinflammatory innate signaling and a defective adaptive response, insufficient to kill the virus or prevent spread (105), thus failing to abort innate immune activation. This review has presented evidence that IRF5 is a key “hub” molecule determining the normal balance between innate and adaptive immunity. In what clinical situations, therefore, could IRAK4/IRF5 inhibitors have therapeutic benefit?

Many clinical consequences arise from immune system dysregulation in COVID-19. Importantly, for proposed treatment of “cytokine storm” with an IRAK4 inhibitor, timing, and dose titration are critical—too early, too protracted or too high a dose and the natural host immune response is further blunted. It follows that reliable real–time (blood) biomarkers of IRF5-driven immune activation would be essential to determine the threshold for both commencement and termination of treatment (120, 121). To avoid overdosage, the ideal IRAK4 inhibitor would be quick-acting with short half-life (122, 123). Amongst candidate COVID-19 inflammation biomarkers is IRF5 itself, raising the possibility of studying this key molecule across the whole range of SARS-CoV-2 infection and associated comorbidities (124, 125); already IRF5 is suggested as a novel adipose marker in chronic metabolic inflammation (108) and inflammatory bowel disease (125).

Another practical issue is management of patients on long-term immuno-suppressants: these drugs could be viewed simplistically as raising the threshold for effective adaptive immune activation, particularly with drugs inhibiting T or B cell function. Nevertheless, it is surmised that many patients could reset their immune response appropriately and experience symptomless or mild COVID-19, but in others, nearer the tipping-point, the chances of hyperinflammatory syndome may be significantly increased. As there is no *a priori* reason in these patients to suppose increased viral uptake at the onset, the advised management of COVID-19 has continued to be on accepted lines and routine immuno-suppressants continued unless “cytokine storm” becomes imminent (126, 127).

Similar reasoning may be applied to initial high viral load or prolonged exposure, which could overwhelm adaptive immunity and push the balance toward increased, but less effective, innate immune activation and “cytokine storm.” A related unresolved difficulty is management of chronic COVID-19 symptoms, especially if associated with identifiable chronic inflammation, including neurological sequelae (115). Indeed, the predisposing conditions for hyperinflammatory COVID-19 are likely to overlap with at least some of those responsible for post-infection sequelae. Whether viral persistence occurs is uncertain, but post-infection inflammatory markers suggest ongoing low-grade innate immune activation linked to adaptive immune dysregulation and/or exhaustion (128). Chronic infection promotes the death of protective CD4+ cells through TLR7 and IRF5 (129). Thus, in so-called “long COVID” the perceived imbalance of innate and adaptive immunity may be finely poised and potentially amenable to favorable manipulation, conceivably using IRAK4 or IRF5 inhibition.

Dexamethasone, anakinra and tocilizumab are amongst anti-inflammatory drugs already repurposed for treatment of “cytokine storm.” Although the extent of dexamethasone interaction with the IRAK4-IRF5 axis is not established, IRAK4/IRF5 inhibitors are still likely to provide a more focused approach than the generalized actions of steroids (130). On the other hand, IRAK4/IRF5 inhibitors would have a wider spectrum of action than the IL-1 receptor antagonist, anakinra (131, 132) or IL-6 receptor blocker, tocilizumab. Predictably, there is concern that overuse/prolonged use of steroids as immuno-suppressants could suppress viral clearance (133): by contrast, IRAK4 inhibition is potentially steroid-sparing (134). Latest data indicates significant benefit in severe COVID-19 from tocilizumab, either alone or with dexamethasone (135–137). CXCL8/IL-8 inhibitors are being trialed to reduce neutrophil recruitment (138, 139). However, as proposed here, a better option might be concurrent suppression by just one drug of multiple innate cytokines and chemokines, including IL-1, IL-6 and neutrophil-attractant chemokines (CXCL8 and CXCL5), as would be achievable by an IRAK4 or IRF5 inhibitor. Indeed, in co-cultured RNA-stimulated pDCs and NK cells, IRAK4 inhibition reduced IL-6, CXCL8, CCL3, CCL4, TNF-α, and IFN-γ (140), whereas, raised expression of IRF5 (but not IRF3 or IRF7) in kupffer cells and neutrophils in experimental cholestatic jaundice correlated with increased IL-6, TLR4, TLR7, TLR9, HMGB1, CXCL8, and CCL2, with some evidence of steroid reversibility (141).

Although developed recently, IRAK4 inhibitors are under assessment in psoriasis, whilst in rheumatoid arthritis a completed phase II clinical trial has demonstrated clinical improvement (142). Interestingly, dimethyl fumarate, already of proven clinical efficacy in treating both multiple sclerosis and psoriasis, is not only a direct inhibitor of IRAK4 but also suppresses innate proinflammatory cytokines in pDCs, providing a strong mechanistic rationale for its recently proposed repurposing for COVID-19 “cytokine storm” (143, 144). Low-grade inflammation is common in autoimmunity (145), with an inflammatory signature similar to COVID-19 (146). The therapeutic usefulness of IRF5 inhibitors is yet to be determined (13, 123, 145–148, 175).

Finally, in SARS-CoV-2 vaccine development, an adjuvant stimulating the evolutionary-conserved, IRAK4-IRF5 pathway should be an ideal partner for a SARS-CoV-2 vaccine. IRAK4-IRF5 pathway activators could be included in multi-epitope vaccines (149). Such formulations should promote optimum immune responses and immunological memory (150). Suitable targets would be TLR3, TL7, TLR8, or TLR9 (151–153). Paradoxically, even with highly potent vaccines, the adaptive immune system in vulnerable groups may still fail to respond appropriately because risk factors predicting a poor adaptive immune response to vaccination could be the same as those predisposing to COVID-19 “cytokine storm,” although it is yet to be determined whether this will account for a significant fraction of vaccine failures.

In conclusion a caveat: given that IRF5 is essential for normal immunity and that “cytokine storm” in SARS-CoV-2 infection indicates a failure of adaptive immunity to respond appropriately to enhanced (IRF5-mediated) innate signals, it follows that attempts to stop “cytokine storm” by damping down innate immunity should be combined with, or ideally replaced by, effective SARS-CoV-2 virucidal drugs, another high priority in COVID-19 research (154–156).

## Author Contributions

The author confirms being the sole contributor of this work and has approved it for publication.

## Conflict of Interest

The author declares that the research was conducted in the absence of any commercial or financial relationships that could be construed as a potential conflict of interest.
